# But they move! Vicariance and dispersal in southern South America: Using two methods to reconstruct the biogeography of a clade of lizards endemic to South America

**DOI:** 10.1371/journal.pone.0202339

**Published:** 2018-09-05

**Authors:** Thomas Nathaniel Hibbard, María Soledad Andrade-Díaz, Juan Manuel Díaz-Gómez

**Affiliations:** Instituto de Bio y Geociencias del Noroeste Argentino, Consejo Nacional de Investigaciones Científicas y Técnicas, Universidad Nacional de Salta, Rosario de Lerma, Salta, Argentina; Keele University Faculty of Natural Sciences, UNITED KINGDOM

## Abstract

This study aims to identify events that modeled the historical biogeography of *Phymaturus*, using three methodologies: Spatial Analysis of Vicariance (VIP), Statistical Dispersal-Vicariance Analysis (S-DIVA), and Bayesian Binary Method MCMC (BBM). In order to assign areas for the Dispersal-Vicariance and the BBM analyses, we preferred not to use predefined areas, but to identify areas defined via an endemism analysis of *Phymaturus* species. The analyses were conducted using the same basic topology, which we obtained by constructing a metatree with two recent phylogenies, both morphology and molecular-based. This topology was also used to obtain time divergence estimates in BEAST, using more outgroups than for the metatree in order to get more accurate estimates. The S-DIVA analysis based on the metatree found 25 vicariance events, 20 dispersals and two extinctions; the S-DIVA analysis based on the BEAST tree yielded 30 vicariance events, 42 dispersal events and five extinctions, and the BBM analysis yielded 63 dispersal events, 28 vicariance events and 1 extinction event. According to the metatree analysis, the ancestral area for *Phymaturus* covers northern Payunia and southern Central Monte. A vicariant event fragmented the ancestral distribution of the genus, resulting in northern Payunia and southern Central Monte as ancestral area for the *P*. *palluma* group, and southern Payunia for the *P*. *patagonicus* group. The analysis based on the BEAST tree showed a more complex reconstruction, with several dispersal and extinction events in the ancestral node. The Spatial Analysis of Vicariance identified 41 disjunct sister nodes and removed 10 nodes. The barrier that separates the *P*. *palluma* group from the *P*. *patagonicus* group is roughly congruent with the southern limit of the *P*. *palluma* group. The ancestral range for the genus occupies a central position relative to the distribution of the group, which implies that the species must have migrated to the north (*P*. *palluma* group) and to the south (*P*. *patagonicus* group). To answer questions related to the specific timing of the events, a molecular clock for *Phymaturus* was obtained, using a *Liolaemus* fossil for calibration. The present contribution provides a hypothetical framework for the events that modeled the distribution of *Phymaturus*.

## Introduction

Vicariance is crucial in the speciation process [1,2]. Even though several other biogeographic processes (duplication, extinction, and dispersal) are also important, vicariance has most largely contributed to the generation of the current distribution patterns. The number of historical biogeography studies based on phylogenetic studies is increasing [3–7]. Molecular phylogenetic studies date the time at which different speciation events occurred [8,9]. Despite all these advances, few studies have attempted to provide the specific location of events through a spatial analysis of distribution.

The lack of these types of studies promoted the development of a new method for reconstructing biogeographic history, which focuses on finding vicariant events rather than just searching for an ancestral area [10]. The Spatial analysis of Vicariance, implemented in Vicariance Inference Program (VIP), uses distributional data in a phylogenetic context. This program identifies sister nodes with disjunct distributions (allopatric/vicariant) and detects possible barriers [10]. The program also performs a heuristic search in which disjunct distributions are obtained by removing the distribution of overlapping taxa. To do so, a cost is assigned to each pair of sister nodes with overlapping distributions and to each removed node. There are no clear guidelines as to the interpretation of these removals; one possibility is to consider them as a dispersal event occurring at the node or its descendant [10]. On the other hand, DIVA (Dispersal-Vicariance Analysis) uses predefined areas, and current taxa are assigned to an area according to their distribution. The program then assigns costs to events of dispersal and vicariance in order to discover the ancestral area at each node, working under a parsimony assumption. S-DIVA (Statistical DIVA) and BBM analyses are methods implemented in RASP (Reconstruct Ancestral State in Phylogenies) [6,11] based on the algorithm used by DIVA; however, unlike DIVA, they calculate probabilities of different ancestral areas at each node.

The phylogenetic history and the distributional patterns of the genus *Phymaturus* provide an interesting example to identify possible vicariance events and geographical barriers that caused the evolutionary radiation of this South American genus. *Phymaturus* is a member of the family Liolaemidae, along with *Ctenoblepharys* and *Liolaemus* [12–14]. Liolaemidae is one of the most diverse groups of lizards in terms of species richness and habitat diversity. *Phymaturus* includes 48 species [15–18] and was classified as Vulnerable in the last categorization of reptiles and amphibians of Argentina (these categories are not equivalent to the UICN categories) [19]. Liolaemidae is a widely distributed family in Argentina, Bolivia, Chile, Peru, the sandy coasts of Uruguay, and southeastern Brazil [20–23]. *Phymaturus* species are primarily characterized by their restricted distributions, with marked endemism in the arid region of southwestern South America (Andes, Patagonia highlands and Puna) [24,25]. Moreover, these species have unique anatomical, physiological and ecological characteristics, which make them especially dependent on a particular environment: rocky outcrops that they use as crevices. [24,26–30]. These outcroppings are sometimes isolated and surrounded by areas where these lizards are not present.

In recent years, several phylogenetic hypotheses have been proposed for the genus [31,32]. These hypotheses are congruent with earlier proposals of two monophyletic groups for the genus: (1) *Phymaturus palluma* group, distributed from Catamarca to Neuquén; and (2) *P*. *patagonicu*s group, occurring in central and southern Argentine Patagonia. Previous studies have investigated the historical biogeography of the group using specific methodology, such as DIVA, Weighted Ancestral Area Analysis and Fitch optimization; however, those studies included fewer terminals than the ones included in this study [25,33]. In these contributions Central Patagonia was identified as the ancestral area of *Phymaturus*, which is congruent with the proposal of Cei [34]. Díaz-Gómez [25] found that part of Central Patagonia was included in the ancestral area, and Payunia was also found to be part of the ancestral area by two of the three methods used in that study.

This study attempts to explain the current distribution of the genus *Phymaturus* and formulates hypotheses about events that modeled its distribution. Our results may help to recognize events that may have affected the distribution of other organisms present in the study regions, since it is unlikely that vicariance events affect only one group of organisms. Possible similarities may likely be found with groups that have similar habitats. Several other taxonomic groups that are also saxicolous have very similar distributions to that of *Phymaturus* (e.g. lizards, such as the *Liolaemus dorbignyi* and *L*. *elongatus* clades, or the *Diplolaemus* genus; several plant genera, such as *Festuca magellanicus*, *F*. *scabriscula*, *Senecio gnidioides*, *S*. *tricuspidatus*, *Poa spiciformis*, *P*. *subenervis*, and *Perezia calophylla*) [35]. Therefore, the identification of biogeographic events in a particular genus may be the first step to know the biogeographic history of a region. Here, we aim to (1) analyze the distribution of species of the genus *Phymaturus* and (2) compare phylogenetic patterns and identify series of vicariance and dispersion events using different methods (VIP, S-DIVA and Bayesian Binary MCMC). The implementation of these biogeographic tools will allow us to compare the results obtained by the different methodologies. It is important to compare the main events, even though complete congruence among the methods is not expected. Congruence among methods might reveal strong evidence underlying the results. In case of ambiguous results of one of these methods, the others may help clarify those results, thereby complementing one another.

## Materials and methods

### Study area

**Fig 1:**
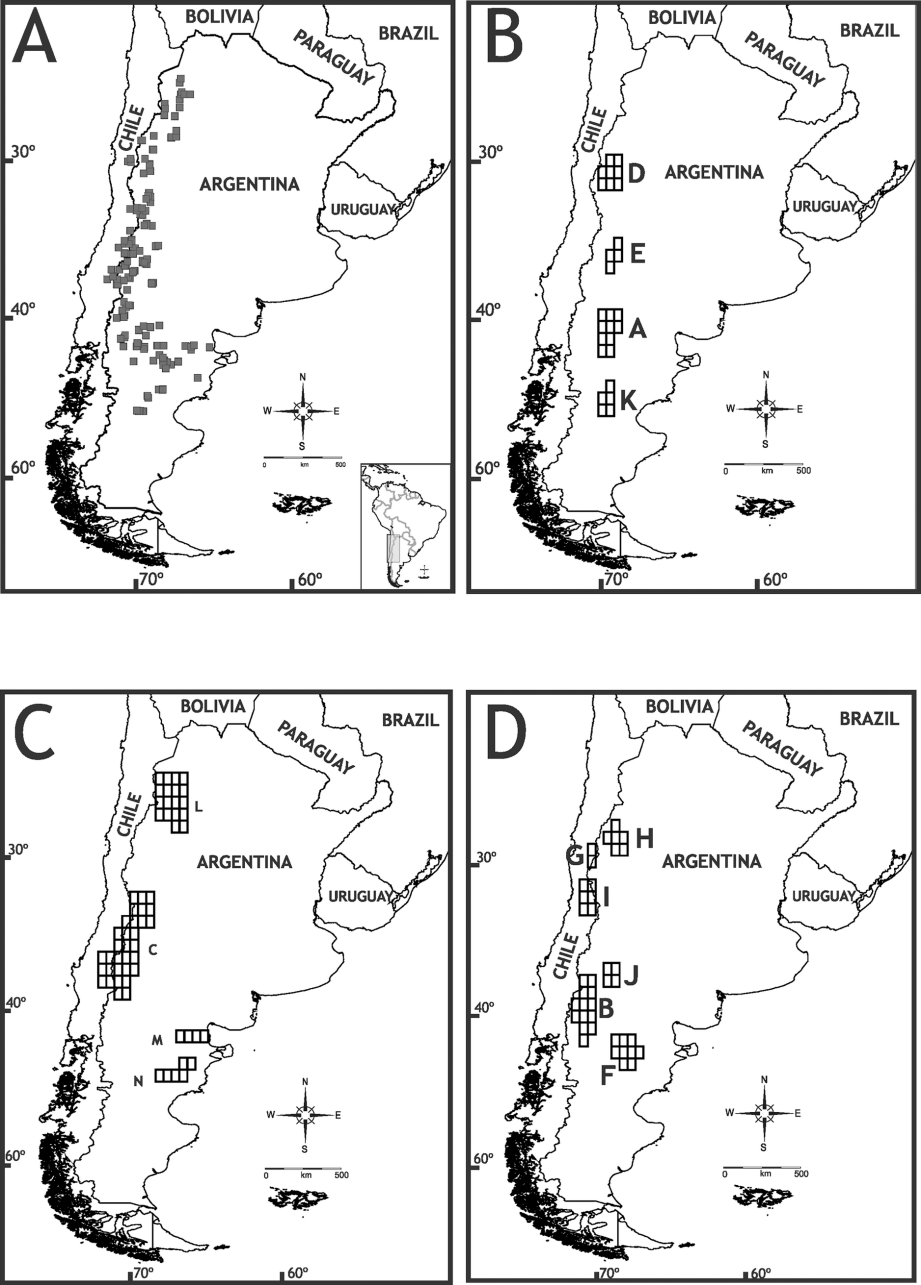
Genus distribution and endemism areas. Distribution records of all known localities for the *Phymaturus* genus (A); endemism areas obtained using NDM software (B-D).

The study area corresponds to the entire distribution of the genus *Phymaturus*, encompassing mountain areas adjacent to the Andes range, from northern Catamarca to Chubut provinces in Argentina, including some localities in Chile (Fig 1A).

### Distributional data

We collected distributional data from 70 terminals, which corresponded to all the 45 species described to date and 26 populations of uncertain taxonomic status (S2 Table). We obtained data from herpetological collections, relevant literature, and online databases (Global Biodiversity Information Facility). Dubious records were checked with help of specialists. The type locality was included for every species as long as it was specific enough for the purposes of this study (e.g. ambiguous type localities such as “Catamarca” were not included).

### Phylogeny

Ancestral area methods and vicariance analysis require a completely resolved phylogeny (i.e. without polytomies) of the study taxa. As the goal of this paper is not to obtain a new phylogenetic hypothesis for *Phymaturus*, we decided to use the most recent and complete phylogenies available for the genus. We used partial phylogenies of the subclades because there is no recent complete phylogeny for *Phymaturus*. The most recent phylogeny for the *P*. *patagonicus* group is the one published by Morando et al. [32], and for the *P*. *palluma* group, the one published by Lobo et al. [31]. Although Morando et al. [32] obtained several trees using different data sets and methodologies, we chose to work with the all-genes concatenated analysis because it had the most complete data set. Additionally, a completely dichotomous tree was obtained via concatenated analysis. Morando et al. [32] also used the complete data set in a BEST analysis, but this tree was not dichotomous and had a low support for many of the nodes. On the other hand, Lobo et al. [31] obtained some polytomies, although they were all located at terminal nodes. We selected one of the most parsimonious trees for this analysis, given that the parsimony analysis included a larger data set and included several terminals for which there were no sequences available (i.e. morphological data). Therefore, in order to select the topologies used, we prioritized well-supported recent phylogenies that included the highest number of terminals. With those two phylogenies we constructed a meta-tree [36] and added outgroups to improve the biogeographic analysis.

Additionally, we performed a BEAST analysis with the aim of comparing results and obtaining a molecular clock. This analysis included all the terminals from the aforementioned phylogenies, plus several outgroups. We obtained sequences for these terminals from GenBank. The list of taxa and accession numbers are provided as Supporting information (S1 Table). The outgroups used for this analysis were: *Liolaemus archeforus*, *L*. *kingii*, *L*. *lineomaculatus*, *L*. *darwinii*, *L*. *eleodori*, *L*. *buergueri*. We selected outgroups including species from the two main clades of *Liolaemus*, *Liolaemus sensu stricto* (Chilean group) and *Eulaemus* (Argentine group). We included more outgroups than the metatree analysis to improve the estimation of divergence times.

### Divergence time estimation

Age of nodes and substitution rates were simultaneously estimated (for both topologies, BI and MP) using Bayesian MCMC (Markov Chain Monte Carlo) approach as implemented in BEAST v2.4.0 [37]. We used the genes that were available in gene bank for most terminals, plus outgroups: 12S and ND4. We used a fossil from *Liolaemus*, the sister genus to *Phymaturus*, to set a mean prior of 20 Myr on the tree height. Since the fossil was assigned to the subgenus *Eulaemus* [38], without specific status, in our study we set it as an outgroup taxon, sister to the species members of the *Liolaemus sensu stricto* subgenus, which are the focal species. Divergence times in BEAST were estimated according to [39]: (1) a lognormal prior was employed for fossil calibrations [40]; (2) a Yule speciation process with a random starting tree was used for the tree prior; (3) an uncorrelated lognormal distributed relaxed clock (UCLD) model was employed, allowing evolutionary rates to vary along branches within lognormal distributions [41]. Three independent runs, each of 50,000,000 generations, were performed, with sampling every 5,000 generations. The three separate runs were then combined (following removal of 10% burn-in) using Log Combiner v2.0 [37]. Adequate sampling and convergence of the chain to stationary distribution were confirmed by inspection of MCMC samples using Tracer v1.6 [42]. The effective sample size (ESS) values of all parameters were greater than 200, which was considered a sufficient level of sampling. The sampled posterior trees were summarized using Tree Annotator v2.0 [37] to generate a maximum clade credibility tree (maximum posterior probabilities) and to calculate the mean ages, with 95% highest posterior density (HPD) intervals, posterior probabilities and substitution rates for each node. The BEAST topology was visualized with Fig Tree v1.2 [43]. We used priors to constrain the analysis using the main group obtained in previous analysis [32] for *patagonicus* and [31] for*palluma* groups. Since the fossil was assigned to a different group, we used a larger set of outgroup taxa for this analysis to improve accuracy of time estimation.

### Ancestral area analysis

Ancestral area analysis requires that taxa be assigned to well defined areas. We performed an endemism analysis based on a previous work conducted in lizards of the Liolaemidae family [7]. In order to obtain endemism areas for the S-DIVA analysis, we used NDM/VNDM v3.0 [44] with a matrix of *Phymaturus* distributional data. The optimality criterion of NDM/VNDM checks the distribution of all taxa in a data matrix and compares them with given areas (set of cells) that are evaluated heuristically. Then, it weighs a score and assigns it to each species according to the ‘fit’ or congruence of its distribution with the area. The score for any given area will be the sum of the individual scores of the species included in that area. As there is no formal argument to select a ‘better’ cell size or analysis parameters, we tested several of these options. We selected a cell size of 0.50° x 0.75° because it yielded the highest number of *Phymaturus* species included in endemism areas. Grid origins were fixed at x = -72, y = 24.2. The radius sizes used were: to fill: x = 40, y = 40, to assume x = 60, y = 60. Searches for areas of endemism were conducted using the following options: save sets with two or more endemic species, with score of 1.5 or higher; swap one cell at a time; discard superfluous sets; keep overlapping subsets only if 50% of species unique. To improve the support of the areas found, we ran 100 replicates of the analysis. The results are shown through consensus areas that merge the original areas (sets of cells), which share a user-defined percentage of their defining species (i.e., areas with small differences in their composition will be merged). Thus, the resulting consensus area shows cells with maximum, low, and minimal values of endemicity [45]. The consensus was calculated using a cut-off of 30% (percent of species similarity) and including an area in the consensus only if it shared that percentage of similarity with all the other areas in the consensus. Species present in more than one area (either overlapping or not) were considered widely distributed. Species that were not included in any area were assigned to an NDM area by proximity or to a new area (Fig 1B–1D, Table 1).

**Table 1:** Species per endemism area.

Areas	Species
**A**	P. patagonicus sp18, P. patagonicus sp19, *P*. *etheridgei*, P. patagonicus sp20, *P*. *ceii*, *P*. *spectabilis*, *P*. *spurcus*, *P*. *excelsus*
**B**	*L*. *tenuis*, *P*. *querque*, P. patagonicus sp17, *P*. *zapalensis*, P. patagonicus sp16, *P*. *tenebrosus*, *P*. *manuelae*, P. patagonicus sp13
**C**	*P*. *dorsimaculatus*, *P*. *palluma chillan*, *P*. *palluma planchon*, *P*. *vociferator*, P. palluma sp4, P. palluma sp3, P. palluma sp6, P. palluma sp8, *P*. *maulense*, P. palluma sp5, *P*. *damasense*, *P*. *tromen*, P. palluma sp9, *P*. *verdugo*, P. palluma sp10, *P*. *palluma*, *P*. *adrianae*, *P*. *delheyi*
**D**	*P*. *aguanegra*, *P*. *williansi*, *P*. *extrilidus*
**E**	*P*. *roigorum*, *P*. *payuniae*, *P*. *nevadoi*, P. patagonicus sp12
**F**	*P*. *calcogaster*, P. patagonicus sp22, P. patagonicus sp11, P. patagonicus sp15
**G**	*P*. *paihuanense*, *P*. *bibronii*
**H**	*P*. *gualcamayo*, *P*. *punae rioja*, *P*. *punae*
**I**	*P*. *aguedae*
**J**	P. palluma sp7, *P*. *sitesi*
**K**	*L*. *kingii*, P. felixi a, P. felixi b, *P*. *indistinctus*
**L**	*L*. *pseudoanomalus*, *P*. *fiambala*, *P*. *mallimaccii*, *P*. *antofagastensis*, *P*. *denotatus*, *P*. *laurenti*
**M**	*P*. *somuncurensis*, P. patagonicus sp21
**N**	*P*. *patagonicus*, P. patagonicus sp14

List of species recovered in each of the endemism areas used in DIVA.

For Ancestral Area Analysis we used DIVA (Dispersal-Vicariance Analysis) and BBM (Bayesian Binary Method) implemented in RASP. DIVA uses a three-dimensional cost matrix that assigns different costs to events (extinctions, dispersals and vicariance) in order to minimize dispersal events. Under this approach, vicariance events have no cost, whereas dispersals and extinctions have a cost of one per area unit added to the distribution. The optimal reconstruction(s) are those requiring the minimal number of dispersal events. In this paper we applied a new implementation of DIVA, called S-DIVA [6]. This tool determines statistical support for ancestral range reconstructions using a novel method, the S-DIVA value. In S-DIVA, the frequencies of an ancestral range at a node in ancestral reconstructions are averaged over all trees. In addition, each alternative ancestral range at a node is weighted by the frequency at which the node occurs. The S-DIVA approach is implemented in the software RASP (Reconstruct Ancestral State in Phylogenies) [6], and the module of S-DIVA analysis is modified from source code of DIVA 1.2 [36]. The parameters used for the analysis were: hold = 32767, maxareas = 4, bound = 32767. We only show ‘most likely states’ results, an option available in S-DIVA.

We performed the Bayesian Binary MCMC (BBM) method of biogeographic and ancestral state reconstruction implemented in RASP (Reconstruct Ancestral State in Phylogenies) 2.1b [38]. We used the tree obtained from BEAST (species tree) and published occurrence data for the analyzed species. The BBM analysis was run by applying the model F81 + Γ, and no outgroup was defined. We ran the analysis for 5,000,000 generations, sampled every 1000 generations and with the first 1000 samples being discarded as burn-in.

### Vicariance analysis

We used the Vicariance Inference Program (VIP) in order to discover disjunct (allopatric or vicariant) distributions among sister groups in a phylogenetic context, [10]. VIP assumes that the only evidence left from a speciation process in a geographic context is an allopatric distribution. The VIP analysis was performed using a grid of 0.5° x 0.5° and a maximum fill of 1, and the barrier was represented by Voronoi lines [46]. The heuristic search was made using the following settings: the cost of removal was set at 1.75, an overlap of up to 10% was allowed and, the “partial removal” option was not allowed. The search was set at 5000 iterations, holding 30 trees per iteration. No annealing was used, and a “full sector search” was made, with a sector size of 20. The options: Pages’ heuristics and Flip nodes were implemented.

Since there is no defined criterion to choose cell size, we implemented the 0.5°x0.5° size used in a recently published endemism analysis conducted in the Liolaemidae family, to which *Phymaturus* belongs [7]. In that analysis, [7] the authors used different grid cell sizes and evaluated the resulting areas by their consistency with known climatic conditions, and their inclusiveness of endemic species in each area.

## Results

### Divergence time estimation

The divergence time estimates found for the main groups in *Phymaturus* are shown in Table 2.

**Table 2:** Divergence time estimates (in millions of years before present).

Phylogenetic group	Age	95% Confidence interval	Geological era
*Phymaturus*	16.15	41.82–30.5736	Oligocene
*P*. *palluma* group	15.99	7.93–25.43	Miocene
*P*. *vociferator* clade	5.86	2.36–10.91	Pliocene
*P*. *bibronnii* clade	12.01	5.92–19.72	Miocene
Node 100	9.11	4.65–15.07	Miocene
*P*. *roigorum* subclade	3.94	1.52–7.20	Pliocene
*P*. *mallimaccii* subclade	9.11	3.08–9.61	Miocene
*P*. *patagonicus* group	14.86	8.14–22.11	Miocene
Node 130	7.48	1.78–14.84	Miocene
Node 128	14.41	8.14–21.84	Miocene
*P*. *spectabilis* group	.88	0.04–2.39	Plesistocene
Node 126	12.4	6.94–18.15	Miocene
*P*. *indistinctus* group	4.16	1.11–8.24	Pliocene
Node 123	12.01	6.88–18.15	Miocene
Node 119	7.53	4.17–11.80	Miocene
*P*. *payuniae* group	6.15	5.08–14.33	Miocene

Divergence time estimates (in millions of years before present) of some of the main groups of the *Phymaturus* genus.

### Ancestral area analysis

#### Reconstruction on the metatree

**Fig 2:**
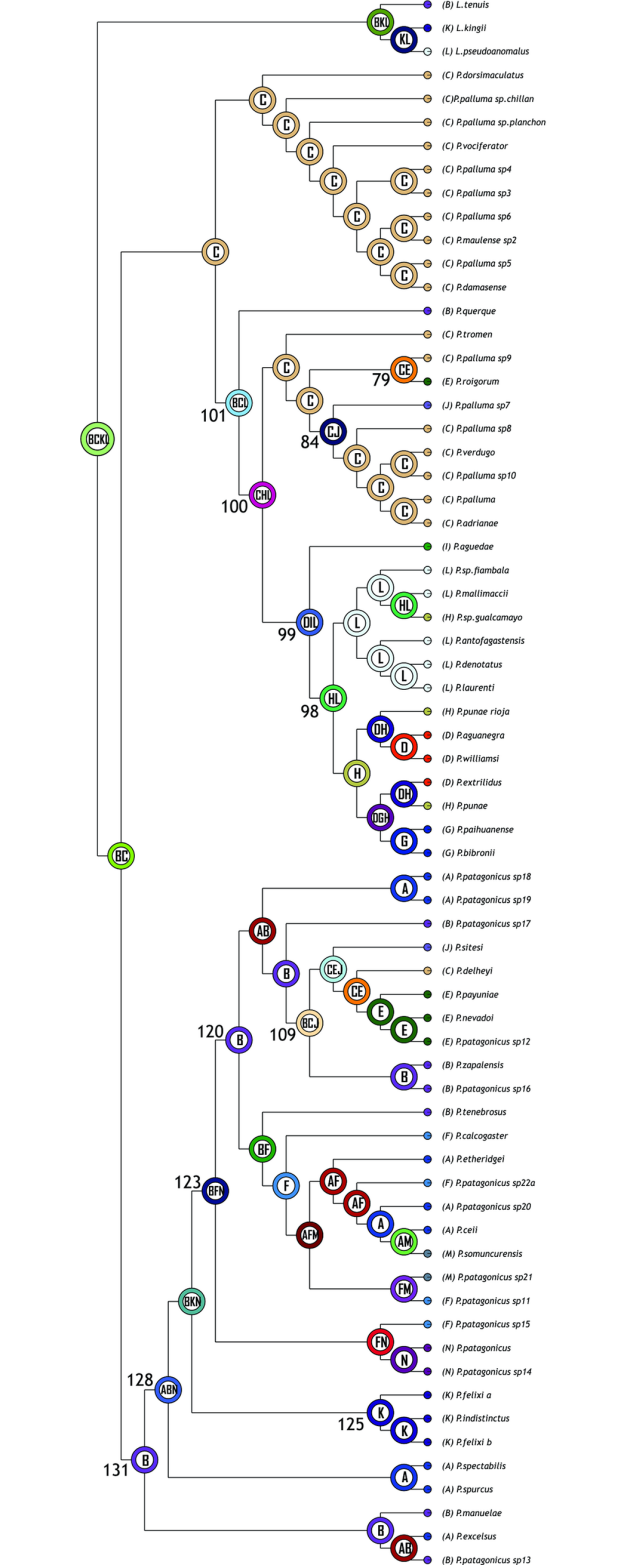
S-DIVA results on the metatree. Each node shows the most likely ancestral area for the node. Numbers next to nodes are used as reference in the text.

**Fig 3:**
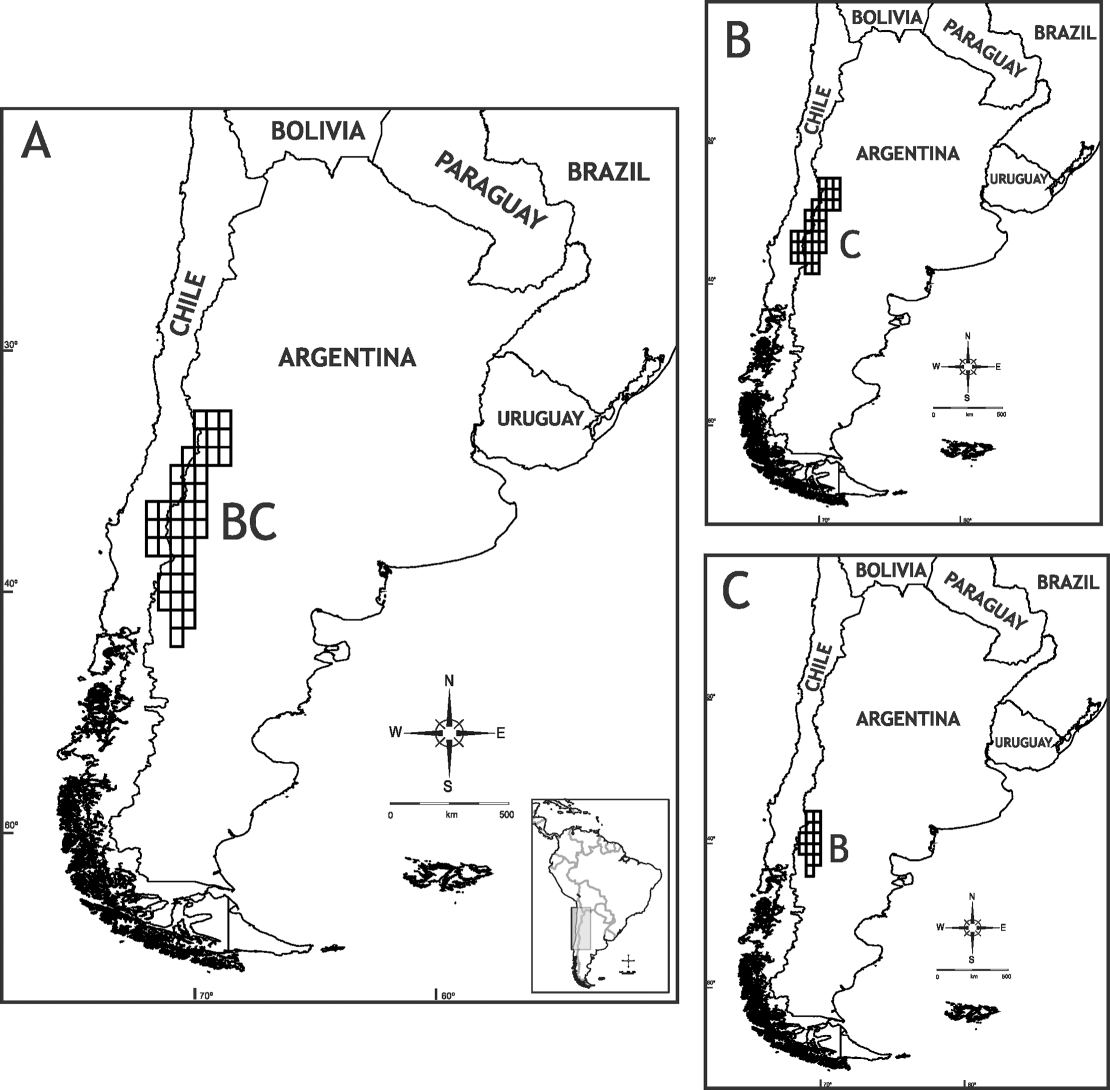
Ancestral areas. Ancestral area for *Phymaturus* (A), *P*. *palluma* group (B), and *P*. *patagonicus* group (C).

The optimal reconstruction found by S-DIVA has 25 vicariant events, 20 dispersals, and 2 extinctions. The complete reconstruction is shown in Fig 2 and the corresponding biogeographic areas are indicated in S3 Table. The ancestral area for *Phymaturus* is BC, which approximately corresponds to Payunia and southern Central Monte, and a vicariant event fragmented this area into the ancestral areas for the *P*. *palluma* and for *P*. *patagonicus* groups (Fig 3).

The ancestral area for the *P*. *palluma* group is C (northern Payunia and southern Central Monte) (Fig 3B). This area is also the ancestral distribution of the *P*. *vociferator* clade (group naming follows Lobo et al. [31]). Two dispersals occurred from the ancestral area of the *P*. *palluma* group: to B and L, resulting in BCL (southern Payunia, Central Monte and Puna) as ancestral area for the *P*. *bibronii* clade. After an extinction event in area B and a dispersal to H (northern Monte and Puna jujeña), the resulting ancestral area for the *P*. *roigorum* group was CHL (norther Payunia, Monte Central and Puna jujeña). In this ancestral area (CHL), several events were suggested: a vicariance event and two extinctions (areas H and L), which resulted in C as ancestral area for the *P*. *roigorum* subclade. C was the ancestral area for all the clades of the *P*. *roigorum* subclade; later a dispersal to J (Auca Mahuida massif, Austral Monte) (node 84), and to E (Payún Matrú and Payún Liso and El Nevado volcanoes) (node 79) was postulated within this group. The suggested ancestral area for the *P*. *mallimaccii* subclade was DIL (Central Monte, Central Chile, northern Monte and Puna jujeña); two extinctions (areas D and I) and dispersal to H (node 98) were suggested there. From the ancestral area formed by HL (northern Monte and Puna jujeña) there is was a dispersal to F, forming the ancestral area for the *P*. *antofagastensis* lineage, FHL (Central Patagonia, northern Monte and Puna jujeña), and an extinction in the ancestral area of the *P*. *punae* lineage, resulting in the loss of area L.

The suggested ancestral area for the *P*. *patagonicus* group was B (Fig 3C). From this area, all other endemism areas are colonized. Initially, areas B and N were colonized, with ABN being the ancestral area for node 128 (i.e., all of the *P*. *patagonicus* terminals except for the *P*. *excelsus* group). The ancestral area for the *P*. *excelsus* group continued to be B. From the area ABN, at node 128, there were vicariance and dispersal events, with the *P*. *indistinctus* groups occupying K (the current distribution of the group), and node 123 dispersing to F (ancestral area for this node: BKN). From this point in the tree again there were vicariance and dispersal events, with node 123 dispersing to F (ancestral area for this node: BFN). At node 123, a vicariance event occurred, with node 125 (P. patagonicus sp15, P. patagonicus sp14, *P*. *patagonicus*) and node 120 having the ancestral areas FN and B, respectively. Interestingly, B was the same ancestral area as the original ancestral area obtained for the *P*. *patagonicus* group, from where all remaining areas of this group were colonized, including those that overlap with the *P*. *palluma* group. Specifically, node 109 (including *P*. *sitesi*, *P*. *delheyi*, *P*. *nevadoi*, P. patagonicus sp12, *P*. *zapalensis* and P. patagonicus sp16) colonized areas C and J (ancestral area of the node: BCJ). On the other hand, the last area to be colonized is area M, which first appears as the ancestral area of the *P*. *somuncurensis* group (ancestral area AFM).

#### Reconstruction on the BEAST tree

**Fig 4:**
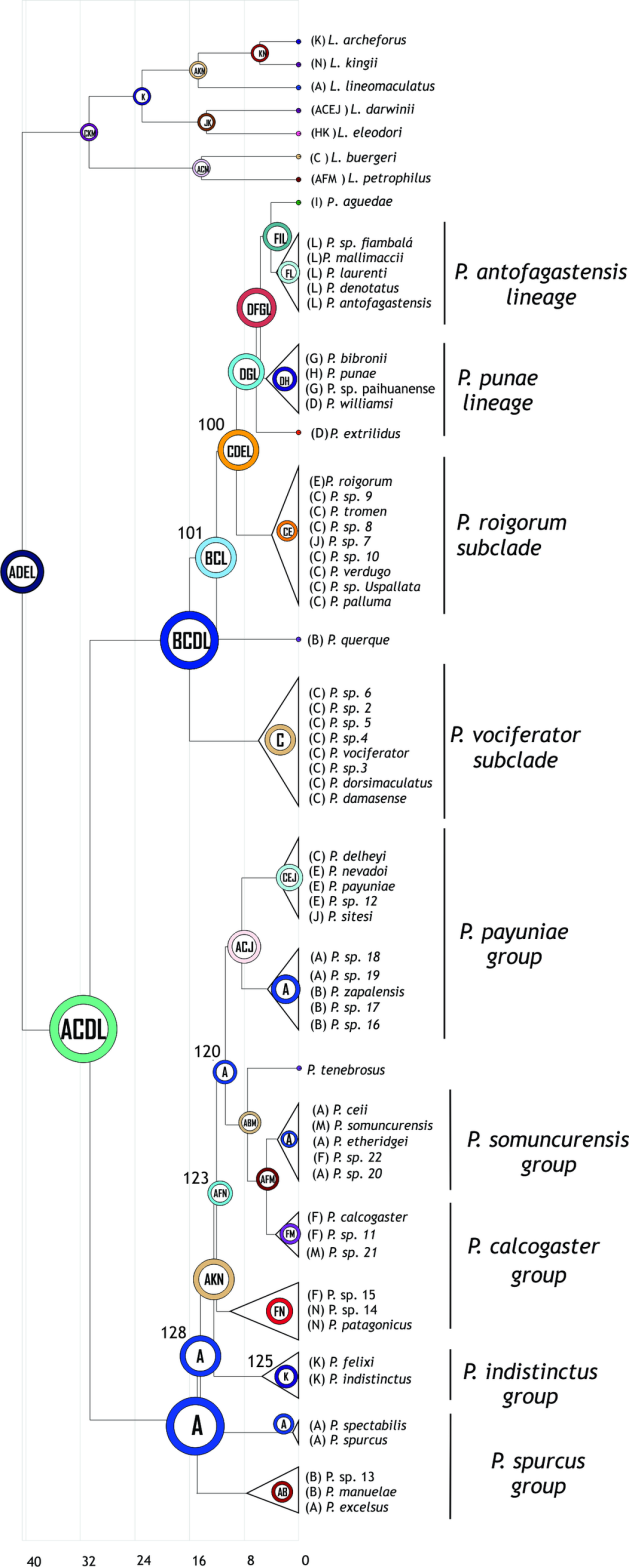
S-DIVA results on the BEAST tree. Each node shows the most likely ancestral area for the node. Numbers next to nodes are used as reference in the text.

The optimal reconstruction found by S-DIVA had 39 dispersals, 31 vicariances and 3 extinctions. The complete reconstruction is depicted in Fig 4. The optimal reconstruction found by S-DIVA on the BEAST tree identified area ACDL (Patagonia, Payunia, Monte and Puna) as the ancestral area for *Phymaturus*, which expands from the Puna to the north (area D) and to northern Central Patagonia to the south (area A). Then, a vicariance event occurred, which split the area: area A (Patagonia), ancestral for *P*. *patagonicus* group, and area CDL, which corresponds to Payunia, Monte and Puna, plus a dispersal to area B. The ancestral area for *P*. *palluma* group is, therefore, BCDL, occupying a larger area within Payunia.

For the *Phymaturus palluma* group, an extinction occurred in area D; there was a speciation event in area C; the *P*. *vociferator* group originated from area C, where all the species of the group originated. On the other hand, *P*. *bibronnii´s* ancestor occupied area BCL (Payunia and Monte). From this point, a vicariance event occurred, which separated *P*. *querque* in area B, from the rest of the *P*. *bibronnii* clade, which also dispersed to areas D (Puna) and E (Tromen massif), yielding the ancestral area: CDEL. Within this group, there were some dispersals, extinctions and vicariances, mainly in the Payuniae region. Then, a vicariance event occurred that split CE (both areas in Payuniae), ancestral for the *P*. *roigorum* subclade, and DL (Monte and Puna). There was also an important dispersal to G, located across the Andes in Chile, so that the suggested ancestral area for the *P*. *mallimaccii* group was DGL. Within this group some dispersals and extinctions occurred in the Puna and Monte regions.

For the *Phymaturus patagonicus* group, the most likely ancestral area recovered was A, the same as the ancestral area for *Phymaturus*. Then there was a split between the basal group (*P*. *excelsus*, *P*. *manulae* and P. sp 13), which dispersed to B (Payunia), yielding AB as the ancestral area for that group, and the rest of the *P*. *patagonicus* group, which remained in A. After this, there was a speciation event in A, which yielded node 130, with A as ancestral area, and the remaining species within *P*. *patagonicus* group, which occupied K and N for the first time for the genus, resulting in the ancestral area AKN (a large portion of Central Patagonia), and reaching the southern limit for the group. Subsequently, there was a dispersal to F and an extinction from K. The suggested ancestral area for the *P*. *indistinctus* group was K, and for its sister group, the ancestral area was AKN (node 123). From this node there was another vicariance event between area A, ancestor for node 116, and area FN, ancestor for the group composed of *P*. *patagonicus*, P. patagonicus_sp14 and P. patagonicus_sp15. Finally, the Payunia areas shared with the *P*. *palluma* group were colonized by the descendants of node 116 (areas B, C, E and J).

#### Reconstruction with the BBM analysis

**Fig 5:**
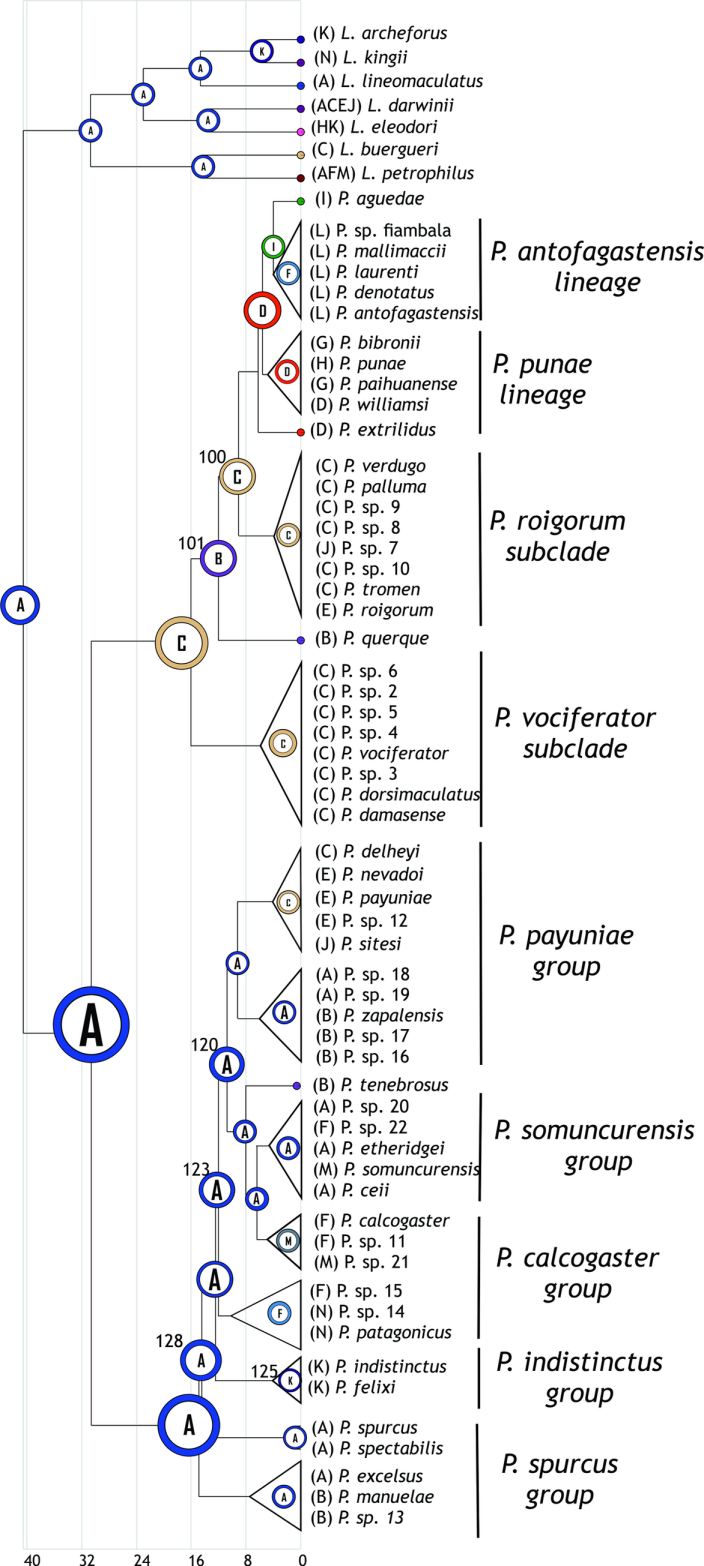
BBM results on the metatree. Each node shows the most likely ancestral area for the node. Numbers next to nodes are used as reference in the text.

The optimal reconstruction found by S-DIVA showed 63 dispersal events, 28 vicariance events and 1 extinction event. The complete reconstruction is shown in Fig 5.

The postulated ancestral area for *Phymaturus* was A. From this area, there was a dispersal event to C, and a posterior vicariance event between A, ancestral for the *P*. *patagonicus group*, and area C, ancestral for the *P*. *palluma* group.

From area C, there was a dispersal event to B, and a vicariance event between B, ancestral for *P*. *bibronnii* clade, and C, ancestral for the *P*. *vociferator* clade. From B, there was a new dispersal to C, and again a vicariance event splitting the basal *P*. *querque*, located in area B, from the rest of the *P*. *bibronii* clade, whose postulated ancestral area was C. From this node, there was a dispersal to D, and later a split, giving rise to the subclades *P*. *roigorum*, ancestral area C, and *P*. *mallimaccii*, area D.

In the case of *Phymaturus patagonicus*, two speciation events occurred in area A at nodes 131 and 128. In this group, the first dispersal event occurred at node 123, where there was a dispersal and a split from K, with K being the ancestral area for the *P*. *indistinctus* group. Then, from node 120, there was a dispersal to F, ancestral for the group formed by *P*. *patagonicus*, P. patagonicus sp. 14, and P. patagonicus sp. 15, and a split from area A, ancestral to node 120. From this node there was another speciation event within area A, ancestral for both the *P*. *payuniae* group and node 119. From the latter, areas M and F were colonized, whereas the *P*. *payuniae* group, dispersed from the ancestral area A to areas C, J, E and B.

### Vicariance Inference Program (VIP)

Without the need of node removal, we obtained 28 disjunct sister pairs in the original reconstruction (grid size 0.5 and a fill of 1). After making the heuristic search, we found 13 new disjunctions using node removals, reaching 41 disjunctions in total. In this search, we recovered two equally costly reconstructions, each removing 10 nodes. In both cases, we found 12 overlapping sister pairs. In order to find disjunctions, VIP ignored the distribution of one of the nodes in the tree, which corresponds to node 123.

**Fig 6:**
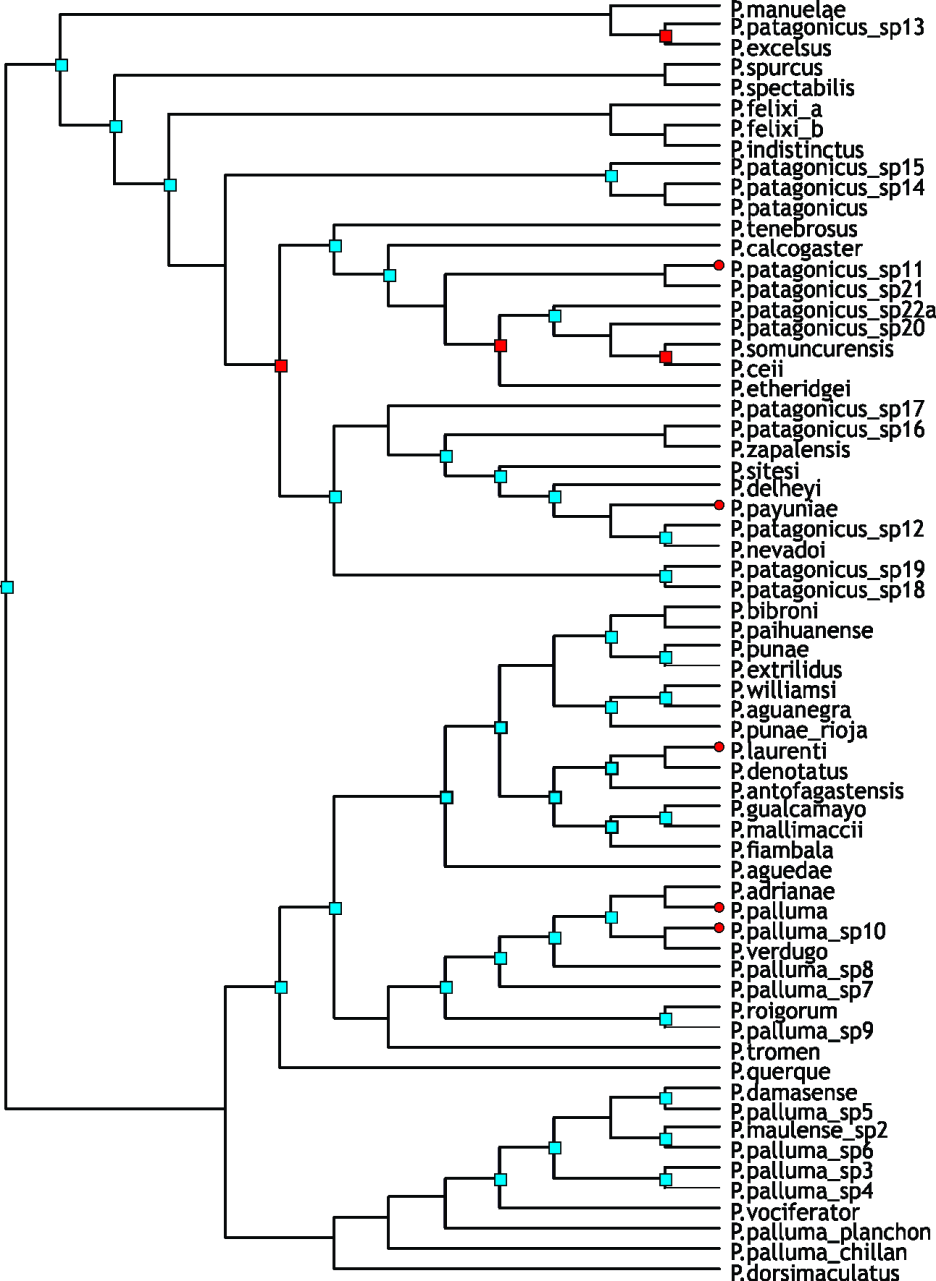
VIP results. Nodes with a square represent a disjunction found by VIP. The distributions of nodes or terminals indicated in red have been ignored.

In the consensus, there were 40 disjunct sister pairs (Fig 6). We found a basal disjunction between the two main groups of *Phymaturus*: *P*. *palluma* group (with a northern distribution) and the *P*. *patagonicus* group (with a southern distribution) (node 132). Both groups overlap approximately between the latitudes 36° and 39°S. The barrier between these two groups postulated by VIP was located approximately at 40° S, i.e. to the south of the distribution of the *P*. *palluma* group (Fig 5A). The location of the barrier coincides roughly with the southern border of the Payunia region (sensu Roig et al. [47]).

**Fig 7:**
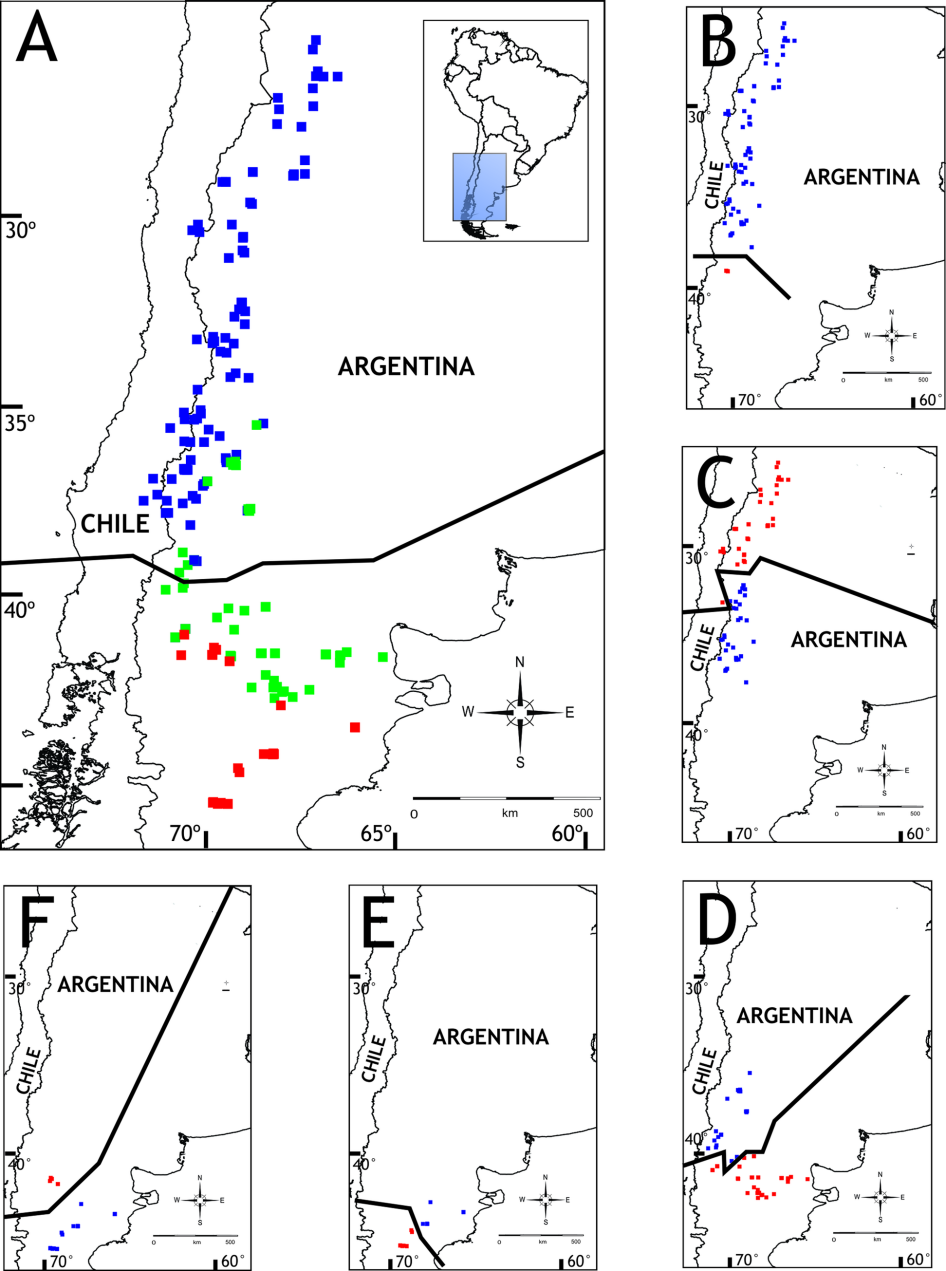
VIP barriers. The approximate barriers found by VIP for *Phymaturus* (A), *P*. *bibronii* group (B), node 100 (C), node 128 (D), node 126 (E), node 120 (F). Blue and red colors determine disjunct sister clades; green dots represent the node whose distribution is ignored.

For the *Phymaturs palluma* group, VIP found a disjunction between *P*. *querque*, basal to the *P*. *bibronii* clade and the rest of this clade (node 101) (Fig 7B). Another disjunction found in the *P*. *bibronii* clade was between the *P*. *roigorum* subclade, occurring mainly the Payunia area, and the *P*. *mallimaccii* subclade, located in the Puna and Monte region of Argentina and Coquimbo region in Chile (node 100) (Fig 7C). Furthermore, inside the *P*. *mallimaccii* subclade, VIP found a disjunction between *P*. *aguedae* located in Coquimbo region in Chile and the rest of the *P*. *mallimaccii* subclade located mainly in Argentine Monte, Puna and Prepuna environments (node 99). Finally, for this subclade, a disjunction was postulated between the *P*. *punae* lineage, more strictly occupying a Puna region, and the *P*. *antofagastensis* lineage, the latter occupying an area located to the north and east of the area occupied by the former, including some northern Monte areas (node 98).

On the other hand, for the *Phymaturus patagonicus* group, *P*. *excelsus* group was found as disjunct from the rest of the *P*. *patagonicus* group (node 131). This disjunction occurred in only two of the four reconstructions recovered. *P*. *excelsus* group occupied an area in the eastern part of the Argentine province of Río Negro, at approximately 41° S latitude. Inside the group of node 131, *P*. *spurcus* group was found to be disjunct from the group formed by *P*. *felixi*, *P*. *indistinctus*, *P*. *patagonicus*, and two populations located in Chubut province (node 128) (Fig 7D). This disjunction did not include node 123, whose distribution was removed by VIP in order to find the disjunction. The barrier detected by VIP is oriented in a SW-NE direction, and runs across the Somuncurá plateau. The southernmost barrier that VIP shown for the *P*. *patagonicus* group was between the *P*. *indistinctus* group and node 125 (actually, it is between these two nodes, since VIP ignored the distribution of node 123) (Fig 7E). The next important disjunction shown was between the *P*. *payuniae* group and the (*P*. *somuncurensis* group + *P*. *calcogaster* + *P*. *tenebrosus*) (node 120) (Fig 7F). In this disjunction, there is a clear separation between a northern group (*P*. *payuniae*) and a southern group (*P*. *somuncurensis*), with the barrier occurring at about 40°30’S.

## Discussion

This study is the first to combine different and complementary biogeographic methods, S-DIVA and BBM, combined with VIP, in an attempt to discover the true biogeographic history of a specific group of endemic lizards. The results of each program show that even though these lizards are highly endemic, they must have had the capacity to disperse at several points in their history.

For the S-DIVA analysis, many studies have used predefined biogeographic areas. For *Phymaturus*, however, those areas tend to overestimate their distribution [48]. For this reason, we chose to perform an NDM-VNDM analysis [44] using only the known distribution points of the genus to obtain the endemism areas. This approach was first used by Díaz-Gómez [25] in an analysis of the ancestral area of the Liolaemidae family. The selection of options used in VIP requires an in-depth explanation, since this program has not been frequently implemented to date. Specifically, there are two options that are largely arbitrary but may influence the results: grid cell size and removal cost. If the chosen cell size is too big, there is risk that the program underestimates the number of disjunctions, since many species that are actually disjunct will appear as occupying the same cell in the grid. On the other hand, if the cell size is too small, there is risk of overestimating the number of vicariances, since some localities will appear to be in different cells of the grid despite being very close in space [10]. Arias et al. [10] recommended using a removal cost higher than 1 (one), but did not recommend any number in particular. We performed several analyses using different costs in order to choose one of these to analyze its results in greater depth. We found that with a cost of 3, the analysis did not remove any nodes, and therefore the results were trivial. In contrast, setting the cost of removal at 1.25 resulted in too many removals, which is not very plausible, given the endemic nature of these species [7]. Results do not vary greatly at removal costs between 1.25 and 3, especially at basal nodes.

Previous studies have focused on the area of origin of the genus (Table 3). Cei [49] postulated an Andean-Patagonian origin of *Phymaturus*. Pereyra [50] postulated the same area of origin, but hypothesized that the southern populations underwent extinction, whereas the northern populations recolonized Patagonia, which is a different scenario to the one we present here. On the other hand, Scolaro [51] proposed that the basaltic plateaus of Patagonia acted as diversification centers for the whole genus. This is also not congruent with our hypothesis, since the groups occupying these plateaus are more terminal in the phylogeny used, and thus our hypothesis is necessarily contrary, with volcanic formations being occupied secondarily, and the ancestral area being in some other place. Some analyses made using quantitative methodology have been published: Díaz-Gómez [33] proposed an area that included Central Patagonia (using WAAA and Fitch analyses), and Central Patagonia plus Cordillera Andina and Central Chile, using DIVA. On the other hand, using DIVA, but a different dataset and endemism areas Díaz-Gomez [25] proposed an ancestral area that comprised Central Chile, Payunia, and northern Patagonia. Our own hypothesis is congruent with the latter, since the ancestral area that we obtained (here denoted BC) occupies a similar range, including Chile and reaching the Central Monte region to the north, and the northern part of Central Patagonia in the south, all along a relatively narrow strip following the Andes range (Fig 3). Moreover, our analysis using VIP shows a barrier at approximately the middle of this ancestral range, suggesting that the ancestor must have at least been present on both sides of that barrier (Fig 7A).

**Table 3:** Previous biogeographic hypotheses for *Phymaturus*.

Group	Paper	Method	Ancestral area
*Phymaturus*	[41]	-	Patagonia
	[42]	-	Patagonia
	[33]	Fitch	Central Patagonia
		WAAA	Central Patagonia
		DIVA	Central Patagonia
	[25]	Fitch	(Prepuna, Payunia, northern Central Patagonia.)
		WAAA	(northwest + center of Central Patagonia)
		DIVA	(Payunia + central-western Patagonia)
*P*. *palluma* group	[33]	Fitch	Valle central (Chile)
		WAAA	Payunia + Valle Central
*P*. *vociferator* clade	[33]	Fitch	Valle Central (Chile)
		WAAA	Payunia + Valle Central
		DIVA	Payunia + Valle Central
*P*. *bibronii* clade	[33]	Fitch	Cordillera Andina
		WAAA	Payunia
*P*. *patagonicus* group	[33]	Fitch	Central Patagonia
		WAAA	Central Patagonia
node 128	[33]	Fitch	Central Patagonia
		WAAA	Central Patagonia
		DIVA	Central Patagonia+Austral Monte+Payunia
node 126	[33]	Fitch	Austral Monte
		WAAA	Austral Monte
		DIVA	Austral Monte+Payunia
node 123	[33]	Fitch	Austral Monte
		WAAA	Austral Monte
		DIVA	Austral Monte+Payunia

**Fig 8:**
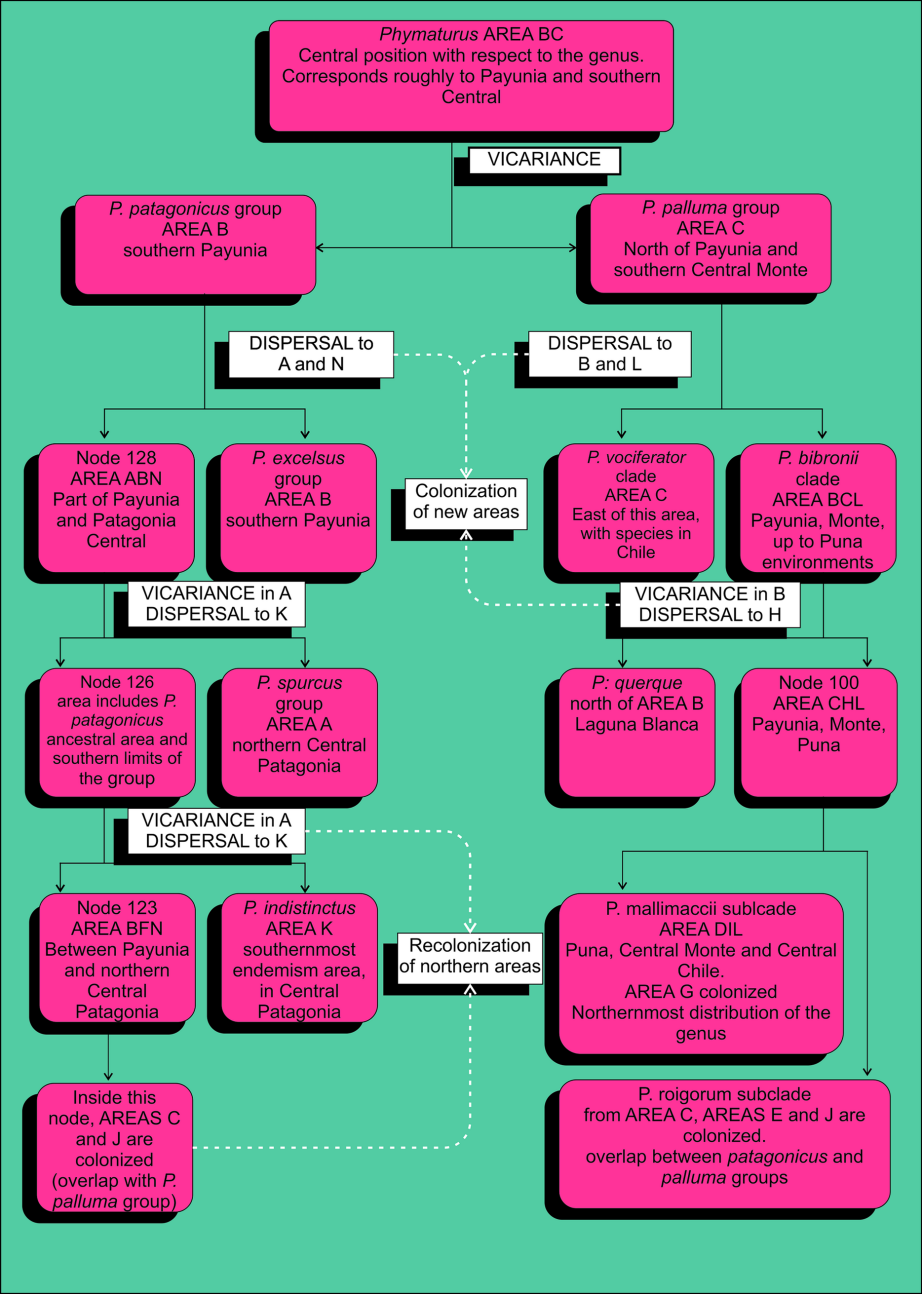
Event flow chart. The main dispersal/range expansion, vicariances and extinctions are shown.

**Fig 9:**
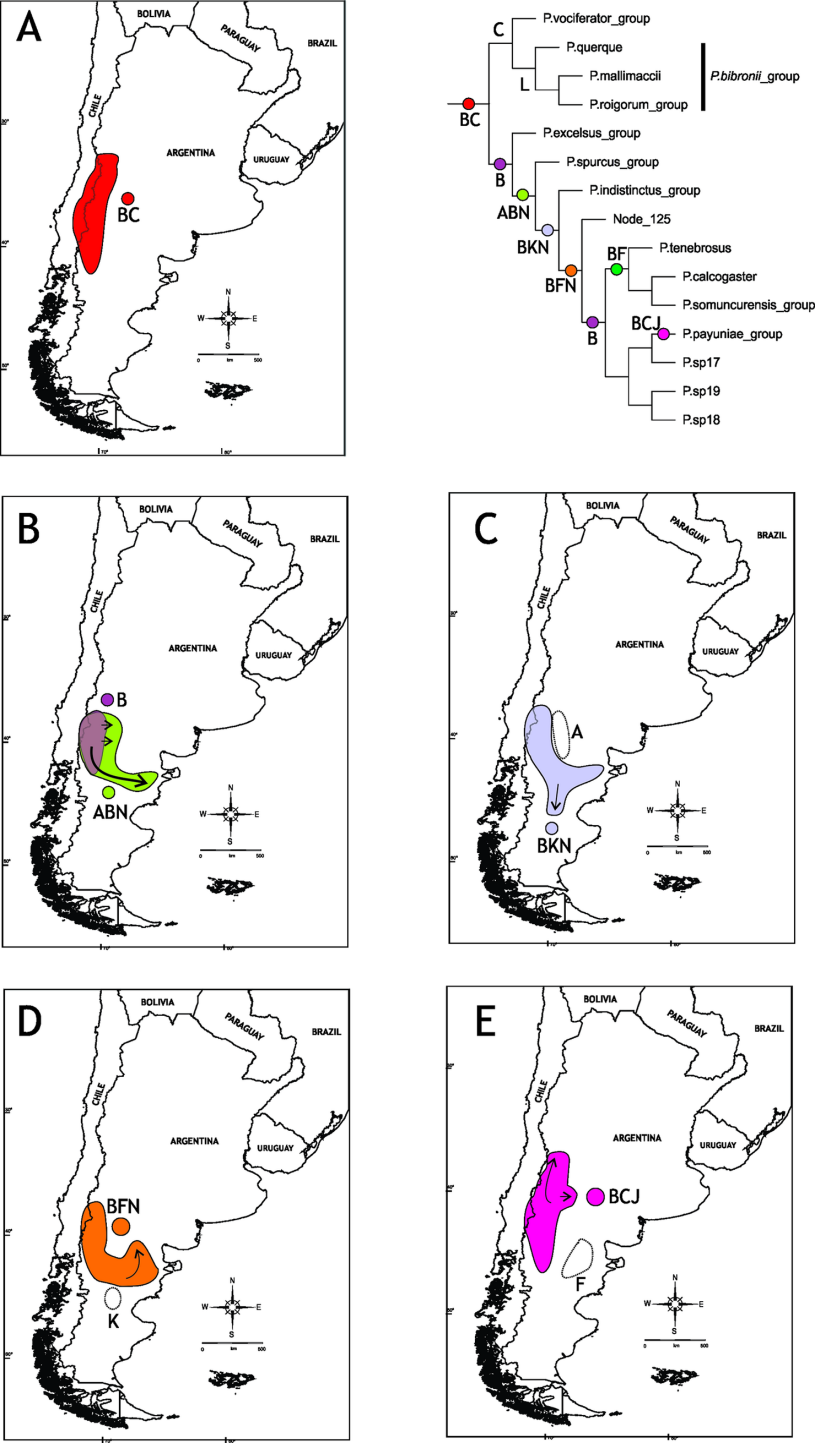
Clade movement. Progressive colonization and extinction for the *Phymaturus patagonicus* group based on the ancestral areas that appear in DIVA. Dotted lines indicate extinction. Areas are shaded following the node colors on the tree.

Several dispersal and vicariance events must have occurred, leading to the current distribution of the genus (Fig 8). In general terms, since the ancestral range occupies a central position relative to the complete genus distribution, the organisms must have migrated to the north (the *Phymaturus palluma* group), and to the south (the *P*. *patagonicus* group) at some point (Fig 9), suggesting some dispersal capacity of these lizards over the course of their history. As mentioned above, this seems unlikely from observing their markedly endemic modern forms. A possible explanation is the formation of rocky outcrops after periods of intense volcanic activity, creating a continuous habitat that allowed dispersal. Later, these outcrops may have been covered by sediments, resulting in the current isolated landscape that we see today.

Regarding the overlap between the groups, four areas are occupied by both *Phymaturus palluma* and *P*. *patagonicus*: area B (northern Patagonia central), C (Payunia and Monte), J and E (both to the east of the Andes mountain range, encompassing recent volcanic formations). VIP ignores the distribution of node 123, which includes all the species occupying the distributions just mentioned; the best way to interpret this result is as a posterior dispersal across the original barrier. Unfortunately, the method does not specify whether the correct way to interpret these results is as a dispersal occurring at the node of interest, or at one of the descendant nodes. On the other hand, S-DIVA provides a more precise interpretation, although it is congruent with VIP in explaining at least part of the overlap. It specifies node 109, a descendant of node 123 (ancestor of the species: *P*. *sitesi*, *P*. *delheyi*, *P*. *payuniae*, *P*. *nevadoi*, P. patagonicus sp12, *P*. *patagonicus and* P. patagonicus sp16), as the first of the ancestors to recolonize area C (Payunia). Yet, although the only conclusion that can be drawn from VIP results is a recolonization by a subclade in the *P*. *patagonicus* group, our analysis of S-DIVA results allows a more complete interpretation, since areas J and E are not part of the original ancestral distribution and in both groups, they are only colonized by derived phylogenetic nodes. It is important to note that in these areas, the species occupy volcanic formations, which provide them with the required habitat, and that these formations are relatively new in geologic time, area J, for example comprising Auca Mahuida Massif, developed 1.7 to 0.88 million years before present, whereas favorable habitats in area E probably date at most to the Pliocene [52]. Accordingly, *de novo* colonization is necessary and fits in the scenario provided by S-DIVA. Thus, the following question arises: which group colonized this area first?

Finally, it is important to note that in this work we have been mostly focused on events happening at basal nodes and with the general pattern arising from the results. We decided to focus on certain significant vicariant events and some necessary movements that must have happened in this genus. It is also central to consider that at this point it is extremely difficult to find specific barriers that may have caused speciation, due to the constantly changing scenarios [53,54], and to the uncertain timing of these events. Previous attempts have been made to identify such barriers. In a phylogeographic analysis, Sercic et al. [55] identified intraspecific barriers that might be common to various groups of vertebrates. Morando et al. [32] hypothesized that the same barriers might have separated species or even groups of species within the *P*. *patagonicus* group. However, these hypotheses should be considered with caution, firstly because the events of the breaks proposed by one study may be very distant in time from the events that separated groups inside the genus, and secondly because there may be other equally likely events that may have not been considered. Finally, the drawn conclusions mostly depend on the topology of the phylogenies chosen [56]. There are different topologies published for the genus. For example, in the BEST analysis provided by Morando et al. [32], the *P*. *spurcus* (*P*. *spurcus*, *P*. *excelsus*, *P*. *spectabilis* and *P*. *manueale*) and *P*. *calcogaster* (*P*. *calcogaster*, *P*. *patagonicus*, *P*. *patagonicus* sp14, *P*. *patagonicus* sp15, *P*. *patagonicus* sp15 and *P*. *patagonicus* sp11) groups are recovered as monophyletic, and the *P*. *indistinctus* group has an unclear position in the phylogeny. As in any other phylogeny-based studies (i.e. ecology, evolution), topology will affect the results. The use of some of the alternative topologies might lead to different biogeographic conclusions. For example, if the *P*. *indistinctus* group (the most southerly distributed group) were recovered as basal to the *P*. *patagonicus* group, the proposed movement for the group would be different, as this would probably force the ancestral area to include some of the areas to the south. Moreover, many of the basal disjunctions obtained in VIP would probably change. However, as previously noted, this is not a problem of the biogeographic analysis *per se*, but a direct result of the phylogenetic hypothesis.

The analyses conducted in this work seemed to be more sensitive to outgroups than to the methodology used, because the results of the BBM and S-DIVA analyses were, to some extent, similar between them, since they shared the same outgroups. For example, in both analyses of the *P*. *patagonicus* group, area A, to the south of the distribution of the genus, consistently appears at each one of the nodes–although this can be seen more clearly in the BBM analysis. Both of these analyses differ from the S-DIVA analysis, which had a different outgroup set, where area B–in this case central to the distribution of *Phymaturus*, and in the biogeographic area: Payunia- is the one that consistently appears at each node. Another key difference is the ancestral area for *Phymaturus*, which is larger in the S-DIVA analyses based on the BEAST topology, possibly because the number of outgroups includes a higher number of areas. This result is also less congruent with the results based on the metatree, which marks a split between groups, at approximately the same position as that where the S-DIVA based on the metatree, marks it–i.e. a split in the Payunia area, between areas B and C- whereas the result of the second S-DIVA analysis is more complex, requiring more events to explain the distribution. Arguably, this could be used as a criterion to select one of the two, but we decided to remain open to both types of results. This level of complexity is maintained throughout the analysis, not only at the basal nodes, as can be seen by the total number of events required in the analysis on the BEAST tree –73–, and the number of events required in the analysis on the metatree –47.

In this paper we used different methods to hypothesize the ancestral area for *Phymaturus* and the main events that modeled its distribution. Moreover, we were able to date several of these events, which in turn can be confronted with geomorphological and paleoclimatic information. As in any other study that uses phylogenies, the results can be affected by changes in the phylogenetic hypothesis. Far from being a disadvantage, this situation allows us to generate and contrast hypotheses, and ultimately, advance in the biogeographic knowledge of this important group of lizards and this region.

## Supporting information

S1 Table GenBank accession numbers fort the terminals included in the BEAST analysis. (XLSX)

S2 Table Number of records of species of the genus *Phymaturus*. (DOCX)

S3 Table Area units used in the analysis. Political and biogeographic correspondence of areas used in S-DIVA.
(DOCX)
