# Supplementary material for: But they move! Vicariance and dispersal in southern South America: Using two methods to reconstruct the biogeography of a clade of lizards endemic to South America
Source: PLoS One. 2018 Sep 5;13(9):e0202339. doi: 10.1371/journal.pone.0202339 (PMC6124713; doi:10.1371/journal.pone.0202339)
Supplement: S2 Table — (DOCX) [file pone.0202339.s002.docx]

S1 Table. Number of geographical records for each species.

| **Species** | **Records** |
| --- | --- |
| *Phymaturus agilis* | 1 |
| *Phymaturus aguadae* | 1 |
| *Phymaturus aguanegra* | 1 |
| *Phymaturus alicahuense* | 1 |
| *Phymaturus antofagastensis* | 3 |
| *Phymaturus bibronii* | 2 |
| *Phymaturus calcogaster* | 3 |
| *Phymaturus casposo* | 1 |
| *Phymaturus castillensis* | 2 |
| *Phymaturus ceii* | 2 |
| *Phymaturus damasense* | 1 |
| *Phymaturus darwini* | 1 |
| *Phymaturus delheyi* | 1 |
| *Phymaturus denotatus* | 2 |
| *Phymaturus desuetus* | 1 |
| *Phymaturus dorsimaculatus* | 6 |
| *Phymaturus etheridgei* | 2 |
| *Phymaturus excelsus* | 1 |
| *Phymaturus extrilidus* | 4 |
| *Phymaturus felixi* | 1 |
| *Phymaturus indistinctus* | 6 |
| *Phymaturus laurenti* | 5 |
| *Phymaturus mallimaccii* | 4 |
| *Phymaturus manuelae* | 1 |
| *Phymaturus maulense* | 1 |
| *Phymaturus nevadoi* | 1 |
| *Phymaturus paihuanense* | 1 |
| *Phymaturus palluma* | 14 |
| *Phymaturus palluma_chillan* | 1 |
| *Phymaturus palluma_fiambalá* | 1 |
| *Phymaturus palluma_larioja* | 1 |
| *Phymaturus palluma_sp3* | 1 |
| *Phymaturus palluma_sp4* | 1 |
| *Phymaturus palluma_sp5* | 1 |
| *Phymaturus palluma_sp6* | 6 |
| *Phymaturus palluma_sp7* | 1 |
| *Phymaturus palluma_sp8* | 1 |
| *Phymaturus palluma_sp9* | 2 |
| *Phymaturus palluma_sp10* | 1 |
| *Phymaturus patagonicus* | 4 |
| *Phymaturus patagonicus_sp11* | 9 |
| *Phymaturus patagonicus_sp12* | 1 |
| *Phymaturus patagonicus_sp13* | 1 |
| *Phymaturus patagonicus_sp14* | 1 |
| *Phymaturus patagonicus_sp15* | 1 |
| *Phymaturus patagonicus_sp16* | 1 |
| *Phymaturus patagonicus_sp17* | 1 |
| *Phymaturus patagonicus_sp18* | 1 |
| *Phymaturus patagonicus_sp19* | 1 |
| *Phymaturus patagonicus_sp20* | 1 |
| *Phymaturus patagonicus_sp21* | 7 |
| *Phymaturus patagonicus_sp22* | 1 |
| *Phymaturus payuniae* | 3 |
| *Phymaturus punae* | 1 |
| *Phymaturus querque* | 2 |
| *Phymaturus roigorum* | 6 |
| *Phymaturus sitesi* | 3 |
| *Phymaturus somuncurensis* | 3 |
| *Phymaturus sp. gua* | 2 |
| *Phymaturus sp. pla* | 1 |
| *Phymaturus sp. uspallata* | 6 |
| *Phymaturus spectabilis* | 2 |
| *Phymaturus spurcus* | 3 |
| *Phymaturus tenebrosus* | 2 |
| *Phymaturus tromen* | 1 |
| *Phymaturus verdugo* | 2 |
| *Phymaturus videlai* | 1 |
| *Phymaturus vociferator* | 1 |
| *Phymaturus williamsi* | 1 |
| *Phymaturus zapalensis* | 5 |
