# Supplementary material for: But they move! Vicariance and dispersal in southern South America: Using two methods to reconstruct the biogeography of a clade of lizards endemic to South America
Source: PLoS One. 2018 Sep 5;13(9):e0202339. doi: 10.1371/journal.pone.0202339 (PMC6124713; doi:10.1371/journal.pone.0202339)
Supplement: S3 Table — Political and biogeographic correspondence of areas used in S-DIVA. (DOCX) [file pone.0202339.s003.docx]

S2 Table. Political and geographical references to areas used in S-DIVA.

| **AREA** | **POLITICAL CORRESPONDENCE** | **BIOGEOGRAPHICAL CORRESPONDENCE** |
| --- | --- | --- |
| **A** | southeastern Neuquén, reaching south of Rio Negro, Argentina | northern Central Patagonia |
| **B** | western Neuquén, reaching south of Rio Negro, Argentina | southern Payunia |
| **C** | northwestern Neuquén, southern of Mendoza, Argentina. Región del Maule, southern Bío Bío, Chile | northern Payunia + and southern Central Monte |
| **D** | western San Juan, Argentina | Central Monte |
| **E** | south central Menoza, Argentina | northeastern Payunia |
| **F** | center and north of Chubut, southern Rio Negro, Argentina | Central Patagonia |
| **G** | northern part of IV región, Chile | Coquimbo |
| **H** | western La Rioja and northern tip of San Juan | northern Monte, Puna Jujeña |
| **I** | Metropolitan region, Chile | Central Chile |
| **J** | northeastern Neuquén, Argentina | Austral Monte |
| **K** | southcentral Chubut, Argentina | Central Patagonia |
| **L** | western Catamarca, center and north of La Rioja, Argentina | Puna Jujeña |
| **M** | southeastern Rio Negro | Austral Monte, Central Patagonia |
| **N** | eastcentral Chubut, Argentina | Central Patagonia, Austral Monte |
